#  Eyes Wide Shut: Gaze dynamics without vision, Symposium 6 at the 20th European Conference on Eye Movement Research (ECEM) in Alicante, 21.8.2019. 

**DOI:** 10.16910/jemr.12.7.12

**Published:** 2019-11-25

**Authors:** Martinez-Conde Martinez-Conde, Bradley Buchsbaum, Fatema Ghasia, Freek van Ede, Stephen L. Macknik

**Affiliations:** State University of New York, Downstate Medical Center, USA; Rotman Research Institute, Canada; (Cleveland VA Medical Center, USA; University of Oxford, UK; State University of New York, Downstate Medical Center, USA

**Keywords:** microsaccades, imagination, visual fields deficit, neural prosthetics

## Abstract

The human ability for visualization extends far beyond the physical items that surround us. We are able to dismiss the constant influx of photons hitting our retinas, and instead picture the layout of our kindergarten classroom, envision the gently swaying palm trees of our dream vacation, or foresee the face of a yet-to-be-born child. As we inspect imaginary objects and people with our mind’s eye, our corporeal eyeballs latch onto the fantasy. Research has found that our eyes can move as if seeing, even when there is nothing to look at. Thus, gaze explorations in the absence of actual vision have been reported in many contexts, including in visualization and memory tasks, and perhaps even during REM sleep. This symposium will present the manifold aspects of gaze dynamics in conditions when the visual input is impoverished or altogether absent. Presentations will address the characteristics of large and small eye movements during imagined and remembered scenes, the impact of visual field deficits on oculomotor control, and the role of eye movements in the future development of neural prosthetics for the blind.

**Video stream:** https://vimeo.com/365522806

